# Use of locally delivered dequalinium chloride in the treatment of vaginal infections: a review

**DOI:** 10.1007/s00404-015-3914-8

**Published:** 2015-10-27

**Authors:** Werner Mendling, Ernst Rainer Weissenbacher, Stefan Gerber, Valdas Prasauskas, Philipp Grob

**Affiliations:** German Center for Infections in Gynecology and Obstetrics, Wuppertal, Germany; Hospital Daler, Fribourg, Switzerland; Medinova AG, Zurich, Switzerland

**Keywords:** Dequalinium chloride, Vaginal infections, Vulvovaginal candidosis, Aerobic vaginitis, Vaginal tablets

## Abstract

**Background:**

Vaginal infections are responsible for a large proportion of gynaecological outpatient visits. Those are bacterial vaginosis (BV), vulvovaginal candidosis (VVC), aerobic vaginitis (AV) associated with aerobic bacteria, and mixed infections. Usual treatments show similar acceptable short-term efficacy, but frequent recurrences and increasing microbial resistance are unsolved issues. Furthermore, vaginal infections are associated with a variety of serious adverse outcomes in pregnancy and generally have a major impact on quality of life. Identifying the correct therapy can be challenging for the clinician, particularly in mixed infections.

**Findings:**

Dequalinium chloride (DQC) is an anti-microbial antiseptic agent with a broad bactericidal and fungicidal activity. Systemic absorption after vaginal application of DQC is very low and systemic effects negligible. Vaginal DQC (Fluomizin^®^vaginal tablets) has been shown to have equal clinical efficacy as clindamycin in the treatment of BV. Its broad antimicrobial activity makes it appropriate for the treatment of mixed vaginal infections and in case of uncertain diagnosis. Moreover, resistance of pathogens is unlikely due to its multiple mode of action, and vaginal DQC provides also a reduced risk for post-treatment vaginal infections.

**Conclusions:**

Vaginal DQC (10 mg) as 6-day therapy offers a safe and effective option for empiric therapy of different vaginal infections in daily practice. This review summarizes the available and relevant pharmacological and clinical data for the therapy of vaginal infections with vaginal DQC and provides the rationale for its use in daily gynaecologic practice.

## Introduction

The human vaginal microbiome is mainly dominated by lactobacilli, depending on the ethnic background of women, and consists of probably more than 250 other different species of bacteria in lower quantities [1, 2]. Although this interactive vaginal ecosystem and its disturbances are not yet fully understood, it is obvious, that it can change to dysbiosis (disturbed homeostasis or unbalanced ecosystem) and demonstrate specific ‘metabolic signatures’ [3] or can be affected by pathogens causing infectious diseases. Moreover each woman possess her own and personal vaginal flora; so any management must consider this individual point of view. Disturbed or abnormal vaginal flora is divided into vaginal infections which are localised and affect mainly to the vagina, nevertheless sometimes disease can spread further to other organs. Most sexually transmitted infections (STI) belong to this latter class of diseases. They do often not affect the vagina itself, but the cervix or the urethra (*Chlamydia trachomatis, Neisseria gonorrhoeae*), while *Trichomonas vaginalis* involves both urogenital parts. Vaginal dysbiosis or infections are usually characterised by an abnormal vaginal discharge and other vaginal signs and symptoms. They can be differentiated into vaginosis, which does not show inflammation signs, and vaginitis (usually candidosis or aerobic infection), which leads to inflammation. Vaginal infections and STI are responsible for a large proportion of gynaecological outpatient visits, from 10 to 20 % of consultations, and are typical in woman of childbearing age. Further important issues with these vaginal infections are the very high rate of recurrences and a variety of associated serious adverse outcomes in pregnancy. Finally, they have a major impact on the quality of life and driving these women exhausted.

Although the knowledge about the bacteria- or yeast-host interactions has increased, there are still open questions regarding the interactions of vaginal lactobacilli and pathogens or the role of biofilm [4–6]. It seems that the local immunity by the vaginal mucosa also plays an important role. The differential diagnosis of the vaginal infections is presented in Table 1, Vaginal infections are highly recurrent and are not only adversely affecting quality of life but are also associated with a variety of serious obstetrical and gynaecological complications (preterm birth, salpingitis, and many others) [5, 7–9]. Three main vaginal infections are well defined: (1) bacterial vaginosis (BV), (2) vulvovaginal candidosis (VVC), and (3) trichomoniasis/*Trichomonas* vaginitis (TV). Additionally, there are other vaginal infections which have not yet been completely characterised and recognised. Especially the ‘intermediate flora’ (based on Nugent score 4–6) and the not well defined ‘mixed flora’ or ‘abnormal vaginal flora’ (AVF) could possibly play a bigger role in the development of preterm birth than expected before [10]. Moreover, aerobic bacteria are also involved in vaginal infections but their role is not yet understood. Based on this knowledge a fourth, still poorly characterised type of vaginal infections has been defined: aerobic vaginitis (AV) [11], which is probably the same entity as desquamative inflammatory vaginitis (DIV), where severe inflammation is observed and complains are more intense [12, 13]. Additionally, mixed vaginitis (MV) and co-infection with coexistence of BV, VVC, and TV, is possible [14], and thus diagnosis and treatment of vaginal infections is not always easy. This situation can be very challenging for the clinician who has to choose the effective treatment according to this diagnosis.

**Table 1:** Differential diagnosis of vaginal infections [11, 49, 109]

	Bacterial vaginosis	Aerobic vaginitis	Mixed/disturbed vaginal flora	Vulvovaginal candidosis	Trichomoniasis
Involved pathogens	Anaerobic bacteria e.g. *Gardnerella* (facultative anaerobic), *Atopobium, Prevotella, Mobiluncus,* etc.	Aerobic bacteria, e.g. *Staphylococci*, *Streptococci*, etc.	Anaerobic and/or aerobic bacteria and/or Candida	*C. albicans* (80–90 %) *C. glabrata* (2–5 %) *C. krusei* (1-2 %)	*Trichomonas vaginalis*
Symptoms	Greyish-white, thin, homogeneous discharge, fishy smell	Persistent yellow-greenish discharge, burning	Increased persistent yellowish thin discharge, burning or itching possible	Itching, burning, dyspareunia, yellowish white “cottage cheese” discharge	Foamy, thin, green yellowish discharge, dysuria, itching, burning
Signs	No redness, no inflammation	Redness, inflammation	Redness and inflammation possible	Redness, inflammation	Irregular spotted redness, local bleeding especially after intercourse
pH	>4.5	>5.0	>4.5	<4.5	≥5.0
Amine (KOH)-test	Positive	Negative	Possible useful	Negative	Possible
Microscopy					
Lactobacilli	Lactobacilli reduced/not present	Lactobacilli reduced/not present	Lactobacilli reduced/not present	Lactobacilli normal or reduced	Lactobacilli normal or reduced
Clue cells	Clue cells present	No clue cells	No/positive clue cells	No clue cells	No clue cells
Leucocytes	Leucocytes normal	Leucocytes strongly increased, toxic	Leucocytes possibly increased	Leucocytes normal or increased	Leucocytes increased
Pathogens	Adherent gram-negative rods, etc.	Coliform bacteria, B-streptococci	Various	*Candida pseudo* hyphae and/or blastospores	*Trichomonas vaginalis*
Culture	Not suitable for diagnosis, low specificity	Limited value	Limited value	Useful, particularly for recurrent VVC	Possible, specific
Diagnosis	3 of 4 Amsel criteria positive or Nugent score 7–10	Symptoms and signs, microscopy or AV-score	Presence of multiple clinical findings	Symptoms and signs, evidence of candida	Symptoms and signs, evidence of *T. vaginalis*
Treatment	Oral or vaginal antiinfective, e.g., metronidazole, clindamycin, and dequalinium chloride Restoration of the vaginal ecosystem	Broad spectrum antiinfective, e.g., dequalinium chloride Corticosteroids Restoration of the vaginal ecosystem	Broad spectrum antiinfective, e.g., dequalinium chloride Restoration of the vaginal ecosystem	Oral or local antimycotic, e.g., clotrimazole, fluconazole, nystatin, dequalinium chloride Restoration of the vaginal ecosystem	Oral antiinfectives (e.g., metronidazole), partner treatment Restoration of the vaginal ecosystem

Treatment options for vaginal infections remain limited and the outcome is often unsatisfactory, particularly regarding the frequent recurrences [15, 16]. This situation is especially frustrating for women but also for the physician. The main treatment objectives are the alleviation of the symptoms, the elimination of pathogens, and eventually the recovery from disturbed to a healthy lactobacilli-dominated vaginal flora. Antibiotics exhibit a specific antimicrobial action which renders them effective drugs for the treatment of infections. To achieve better long-term results, every effort should be made to eliminate factors that predispose an individual to recurrent infection. Maintenance-suppressive regimens of antimicrobial agents and the repetitive application of hydrogen peroxide-producing lactobacilli to restore and support the healthy vaginal flora should be considered as treatment options to prevent recurrences [5, 7, 17–22].

The choice of an anti-infective compound depends on the type of infection: According to various guidelines, 5-nitroimidazoles (e.g., metronidazole, tinidazole) are used for the treatment of BV and TV. VVC is treated with orally or locally administered imidazoles or triazoles (e.g., clotrimazole, miconazole, and fluconazole), polyenes or ciclopiroxolamine. Thus depending on the pathogens involved, different antimicrobial agents (antibiotics, antiseptics, antimycotics, etc.) are used as first line therapy, and controlled trials have shown cure rates of 70–80 % after 4 weeks of treatment [5, 7, 8, 18, 21–31].

For the treatment of BV, anti-infectives with activity against anaerobes are indicated, and routine treatment of sexual partners is usually not recommended [32, 33]. Currently, metronidazole and clindamycin taken orally or applied vaginally are the mainstays of BV therapy. They have different spectra of antimicrobial activity but equivalent efficacy with regard to short-term and long-term cure rates [15, 23, 34–36]. Oral therapy with metronidazole is usually preferred over vaginal application according to the various guidelines; however, due to its metabolism in the liver it is associated with frequent gastro-intestinal side effects [27, 35, 37–39]. Clindamycin is usually recommended in the form of a vaginal cream. There are several disadvantages associated with these two therapies [15]: a significant proportion of patients do not achieve an adequate response [27, 40], recurrence of BV frequently occurs [23, 27, 38], drug resistance may develop [4, 8, 41–43], and there is a risk of post-treatment candidosis [38, 44–46].

VVC is usually treated with topical azoles that are available in a variety of formulations [7, 8, 22, 47]. No evidence exists to suggest that one azole type or formulation results in better cure rates. The cure rates for topical azoles are approximately 80 % [48]. Oral azole formulations also achieve comparable cure rates; however, they have the potential of systemic toxicity, sometimes restricting its use. [7, 8, 22]. Particularly considering recurrence of *Candida* infection and azole resistance, treatment with non-azole agents has its place in practice and might be even more important in the future [8, 21, 25, 49].

The basis of TV therapy remains the 5-nitroimidazole group of drugs—metronidazole and tinidazole [7, 8, 49]. Oral therapy is necessary, because infection of the urethra and periurethral glands provides sources for endogenous relapses. Cure rates of up to 95 % may be achieved when sexual partners are treated concomitantly [7, 8, 49].

Standard treatment for other vaginal infections has not been established yet. For AV, clindamycin or antibacterial agents with activity against aerobic gram-positive and -negative bacteria, such as kanamycin [11, 26, 50, 51] but also corticosteroids [12] are suggested. Alternatively, products containing oestrogen alone or in combination with lactobacilli have been used [52]. For the treatment of MV [14] drug substances with broad spectrum of antimicrobial activity are used or a strategy of two-step therapy might be proposed.

Increasing resistance against metronidazole and clindamycin has been reported by several investigators [40, 42, 43, 53]. So far, true acquired antimicrobial resistance occurring in vaginal infections has not been observed as a major problem in clinical practice [27, 54]. Nevertheless, misuse of the over-the-counter (OTC) antimycotics, as well as widespread use of systemic oral azoles, could result in spread of azole-resistant *Candida albicans*, and even more likely it could lead to an increase in non-albicans *Candida* species with intrinsic azole resistance [54, 55].

In the case of any vaginal infection, the healthy vaginal microflora is usually disturbed, i.e., the number of lactobacilli is dramatically reduced or less beneficial lactobacilli such as *Lactobacillus iners* are dominant, resulting in unstable vaginal microbiome [56–58]. Following anti-infective treatment, local therapy with beneficial probiotic lactobacilli is recommended to support long-term restoration of vaginal flora [59, 60]. Repetitive lactobacilli therapy also has been proposed to normalise the vaginal ecosystem to prevent the recurrences of vaginal infections, especially BV [59, 61, 62].

Although antibiotics are selective and effective anti-infectives, it is nevertheless still necessary and desirable to broaden the therapeutic possibilities [1, 15]. As alternative therapy broad spectrum antimicrobial agents, such as dequalinium chloride (DQC), are used to treat vaginal infections. Typical representatives are phenyl derivates (e.g., chlorhexidine, hexetidine), acids (e.g., salicylic acid), halogens (e.g., povidone-iodine), and quaternary ammonium compounds (e.g., DQC). Less specific antimicrobial agents have the advantage that (1) resistance of pathogenic microorganisms is not to be expected due to its multiple mechanism of action [63–66], (2) they can also be used for mixed vaginal infections because of their broad antimicrobial spectrum, as well as additionally (3) for pre- and post-operative prophylaxis.

The goal of the current article on DQC is to review the pharmacology and clinical evidence to better understand this substance and to provide the rationale for its use in daily clinical practice for the treatment of vaginal infections.

## Pharmacology

**Fig. 1:**
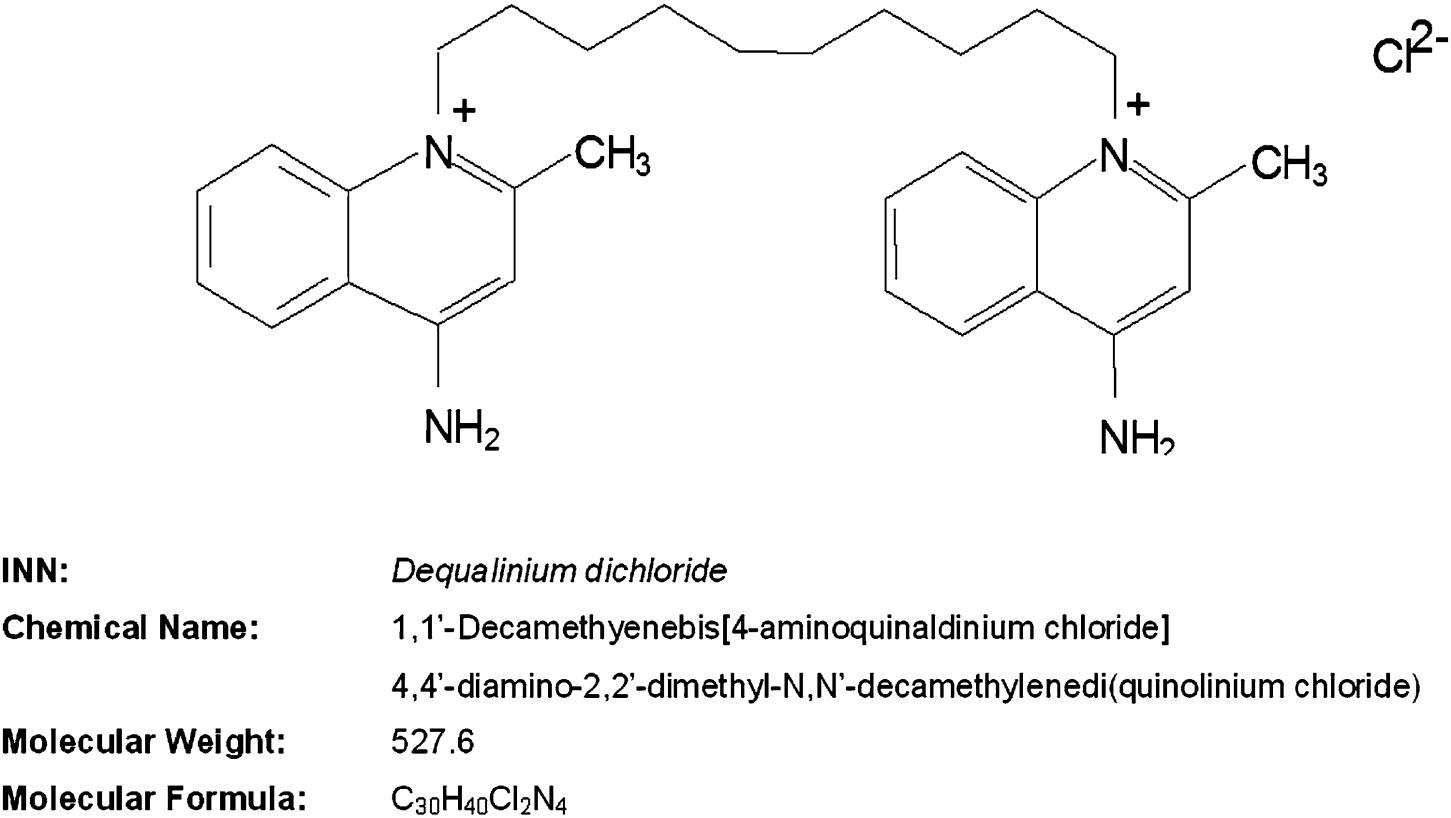
Structure of dequalinium chloride (DQC)

Dequalinium chloride (DQC)—1,1′-(decane-1,10-diyl) bis (4-amino-2-methylquinolinium) dichloride—is a bis-quaternary ammonium compound with a broad spectrum against gram-positive and -negative bacteria, yeasts, and protozoa [67–70] (Fig. 1).

The antimicrobial activity of DQC and quaternary ammonium salts in general, is primarily based on their ability to increase cell permeability with subsequent loss of enzyme activity [66, 70–72]; the substance exhibits a rapid bactericidal and fungicidal action [67].

**Fig. 2:**
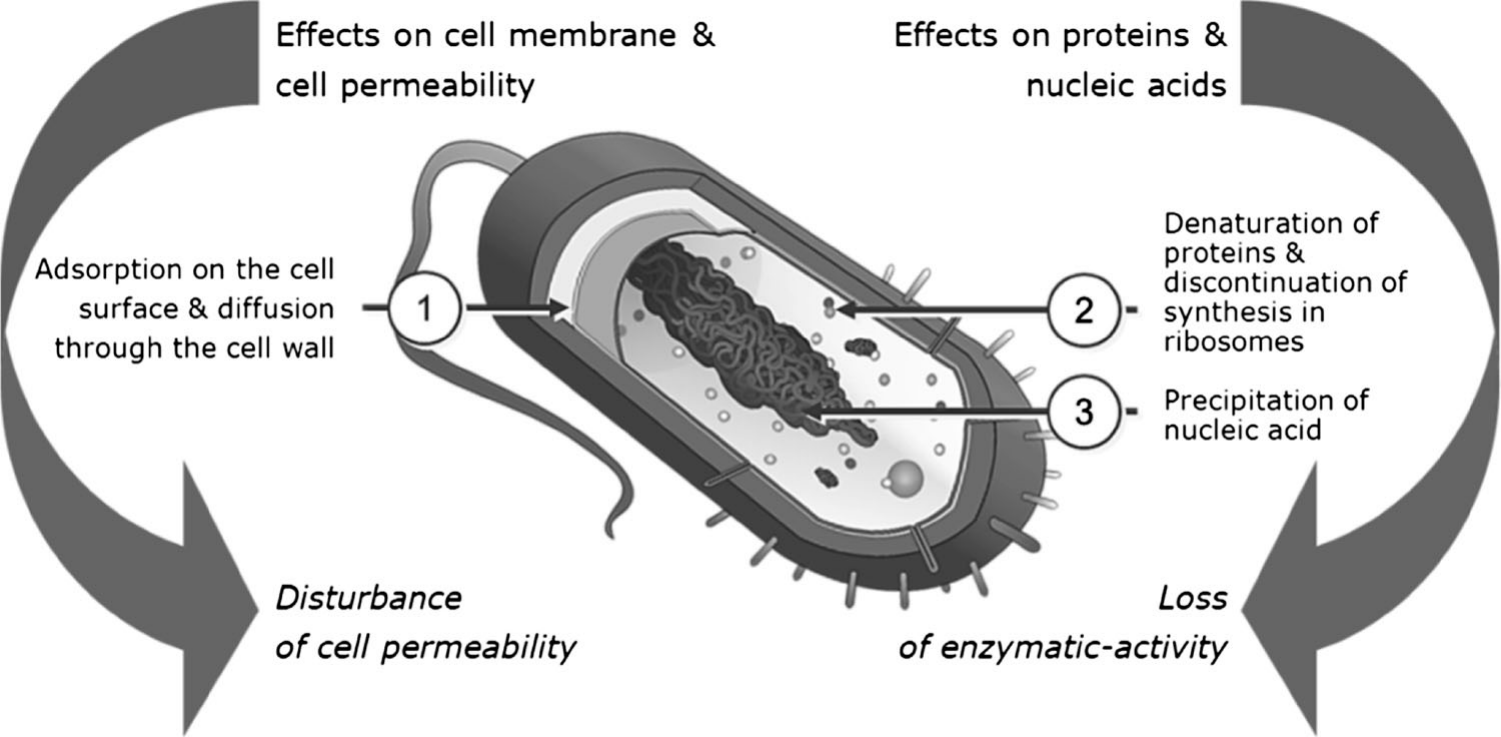
Mechanism of action of dequalinium chloride (DQC)

In detail the mechanism of action of DQC is based on the following mechanisms (Fig. 2):

1. Its effects on bacterial cell permeability are based on (1) the adsorption on the microorganism’s cell surface and diffusion through the cell wall, and (2) binding to the cytoplasmic membrane with subsequent formation of complexes and protein precipitation [65, 66]. The cell membrane might be lysed (depending on the concentration), resulting in perturbed osmotic exchange.
2. The effects on microbial proteins and metabolic reactions after diffusion through the cell wall are caused through (1) denaturation of proteins resulting in inhibition of bacterial cell metabolism, (2) disruption of bacterial energy production through inhibition of glucose metabolism and inhibition of mitochondrial ATP synthesis via inhibition of bacterial F1-ATPase, and (3) termination of protein synthesis at the level of ribosomes [65, 66, 73]. Furthermore effects on bacterial nucleic acids occur such as (1) precipitation of cytoplasmic material with nucleic acids being the most sensitive, and (2) binding to DNA (in vitro) [63, 65, 66].

Disruption of cell permeability and subsequent loss of enzymatic activity is regarded as the primary cause of bacterial cell death after contact with surface-active compounds [65]. The enzymatic inactivation initially might be reversible but becomes irreversible after a longer contact time between DQC and the bacteria. Protein denaturation and inhibition of metabolic reactions are only obtained at doses above the clinical use of quaternary ammonium compounds, and are therefore less relevant for their antimicrobial action.

The antimicrobial activity of DQC has been investigated and demonstrated over the past decades by several investigators [67, 69, 70, 72, 74–80]. An overview of the in vitro antimicrobial activity of DQC is shown in Table 2, DQC has a broad antimicrobial activity against all relevant vaginal pathogens. The bactericidal and fungicidal effect of DQC has been demonstrated to occur within 30–60 min [67]. Della Casa and colleagues demonstrated the in vitro antimicrobial activity of DQC against different pathogens that are relevant for vaginal infections, including anaerobic bacteria (*Gardnerella vaginali*s, *Bacteroides* spp., *Peptostreptococcus* spp., etc.), aerobic bacteria (staphylococci, streptococci, *Escherichia coli*, etc.), *Candida* species (*C. albicans*, *C. glabrata,* etc.) and *Trichomonas vaginalis* [69]. The antimicrobial activity of DQC against *Candida spp.* is comparable with clotrimazole and ciclopiroxolamine [69]. A recent in vitro study has also demonstrated a high susceptibility of *Atopobium vaginae* to DQC, and confirmed that DQC acts bactericidal [80].

**Table 2:** *In vitro* antimicrobial activity of dequalinium chloride (DQC) [66, 67, 69, 74–80]

	Gram stain	MIC_range_^a^ (µg/ml)	MIC_90_^b^ (µg/ml)
Anaerobic bacteria			
*Gardnerella vaginalis*	−/+	2–512	256
*Atopobium vaginae*	−/+	<0.0625–0.5	0.5
*Bacteroides* spp.*/Prevotella* spp.*/Porphyromonas* spp.	**–**	8–512	256; 512
*Peptostreptococci*	**+**	1–32	
*Fusobacteria*	**–**	32–64	
Aerobic gram-positive bacteria			
*Enterococcus faecalis*	**+**	0.2–64	64
*Lactobacillus* spp.	**+**	0.5–8	4
*Staphylococcus aureus*	**+**	0.2–8	1.28; 8
*Streptococcus agalactiae* (group B streptococci)	**+**	2–64	8; 32
*Streptococcus pyogenes* (group A streptococci)	**+**	0.25–20	2
Aerobic gram-negative bacteria			
*Enterobacter* spp.	**–**	3.1–400	
*Escherichia coli*	**–**	1–400	128
*Klebsiella s*pp.	**–**	3.1–400	
*Pseudomonas* spp.	**–**	5–400	
*Serratia* spp.	**–**	3.1–400	
*Proteus* spp.	**–**	20–>1024	
Fungi			
*Candida tropicalis*		0.2–50	
*Candida albicans*		0.2–50	1; 8
*Candida glabrata*		0.2–256	1; 256
*Candida krusei*		128	128
*Aspergillus* spp.		1.6–6.3	
*Trichophyton* spp.		1.56–3.12	
Protozoa			
*Trichomonas vaginalis*		28.8–400	57.6

^a^MIC_range_: minimum and maximum MIC values for certain species

^b^MIC_90_: MIC value at which 90 % of the strains of this species were inhibited

No development of resistance of micro-organisms to DQC has been reported in laboratory studies or clinical trials. Resistance of pathogens is unlikely due to the multiple mode of action of DQC. In fact, multidrug resistance to single quaternary cationic anti-infectives is associated rather with hypersensitivity to compounds containing two quaternary cations, including DQC [81].

For human subjects, published information on quaternary ammonium compounds confirms that the blood levels after oral administration (10–40 mg) are generally low [82–87]. It is expected that absorption is even lower after vaginal application [85], supported by the fact that vaginal application of DQC is very rarely associated with systemic adverse events as demonstrated in clinical studies. In a local repeated dose vaginal tolerability study in rabbits, where daily doses per kg were 2.5–5 times higher than in clinical use, no DQC was detected in the blood. Absorption of DQC in rabbits after vaginal application was below 0.625 % of the administered 5 times higher dose/kg, thus maximum blood levels only in the pg/ml could be expected after vaginal application of DQC in humans. This is consistent with findings in animal toxicology studies, indicating that DQC is absorbed to an extremely small degree (<0.1 %) after oral application [74].

No reproductive toxicity studies have been conducted in animals because of the anticipated low systemic exposure to DQC after vaginal administration. Animal studies with other quaternary ammonium compounds have not shown embryo-foetal toxicity at clinically relevant doses [84]. Preclinical data indicate that DQC is absorbed only to a very small degree after vaginal application, i.e., systemic exposure is negligible [74].

DQC 10 mg vaginal tablet (Fluomizin^®^) has been developed as a directly compressed vaginal tablet to ensure fast disintegration of the tablet and rapid dissolution of the active substance. As soon as the vaginal tablet comes into contact with the vaginal secretion, the tablet begins to disintegrate and DQC is released. After dissolution of the DQC 10 mg tablet in an estimated 2.5–5 ml of vaginal fluid, the DQC concentration is estimated at 2000–4000 μg/ml, assuming negligible absorption. This concentration is four to eightfold higher than the minimal inhibitory concentration (MIC) of the least susceptible isolate (MIC = 512 µg/ml) [66]. An in vivo anti-infective effect is generally obtained if the concentration of the active substance at the site of action is 2–4 times higher than the MIC for 20 min up to 2 h [69].

The broad spectrum antimicrobial activity covering all relevant pathogens for vaginal infections and the negligible systemic absorption are the key factors of DQC to make it suitable for the treatment of most vaginal infections.

## Clinical studies

Local treatment of vaginal infections with DQC is well established. Various clinical studies published over the last 4 decades demonstrate its good clinical efficacy in different vaginal formulations [26, 28, 32, 34, 88–106].

The dose tested for the treatment of vaginal infections ranged from 10 to 50 mg. The immediate release preparations were used with a strength of 10 and 20 mg DQC, whereas controlled release preparation contained 20 or 50 mg of DQC. Treatment duration was usually 3–10 days. The efficacy over the entire dose range was similar and no difference in tolerability was described [26, 28, 32, 34, 88–106]. The medicinal products containing DQC which were tested for vaginal administration in clinical studies are briefly summarised in Table 3.

**Table 3:** Medicinal products containing dequalinium chloride (DQC) reported in the treatment of vaginal infections

Medicinal product	Formulation	References
Dequadin^®^	Vaginal pessary 10 mg	[88, 95, 99]
Dequavagyn^®^	Ovule 50 mg; controlled release for 8–10 days	[34, 96]
Dequavagyn^®^	Cream 20 mg/10 g cream; controlled release for 8–10 days	[34, 91–94, 96, 97, 100]
Oestro-Dequavagyn^®^	Cream 20 mg/10 g cream; controlled release for 8–10 days +0.15 mg estriol/10 g cream	[93, 98]
Oestro-Dequavagyn^®^	Cream 20 mg/10 g cream; controlled release for 8–10 days +2 mg Estriol/10 g cream	[93]
Gyken^®^	Ovule 20 mg	[28]
Eriosept^®^	Vaginal foam 100 mg/100 g	[101]
Fluomizin^®^	10 mg vaginal tablet	[26, 32]

Within clinical studies, more than 3000 women with vaginal infections of different aetiology have been treated with vaginal DQC (Table 4). Clinical studies have been mainly carried out in the treatment of the following vaginal infections: (1) BV, (2) VVC, (3) less characterised aerobic and mixed vaginal co-/infections (‘fluor vaginalis’).

**Table 4:** Overview of clinical studies with dequalinium chloride (DQC)

Publication and design	Subjects and indications	Medication	Efficacy	Safety
*Monopreparation*				
Weissenbacher, 2012 [32] Controlled, comparative	321: BV—321	Fluomizin^®^ vaginal tablets vs. clindamycin 2 % cream vaginal 6 days	At week 1, cure rates were 81.5 % with 10 mg DQC and 78.4 % in clindamycin group. 10 mg DQC vaginal tablets had equal efficacy as a clindamycin vaginal cream. At week 4, cure rates were 79.5 % with 10 mg DQC and 77.6 % with clindamycin, demonstrating that the efficacy of DQC was not inferior to that of clindamycin (significant).	Only ADRs - discharge followed by vulvovaginal pruritus
Radzinsky et al., 2011 [107] Comparative, two-stage therapy	622: BV—409; ‘nonspecific vaginitis’—213	Fluomizin^®^ vaginal tablets 6 days vs. standard anti-infective treatment (afterwards Gynoflor^®^ restoration)	Fluomizin^®^ group – cure 77.8 % (best dynamics observed for disappearance of the ‘clue cells’); standard antiinfective treatment – cure 77.1 % (average).	Not assessed
Dankovich and Gopchuk, 2006 [102] Open, pre-operative prophylaxis	45: Abnormal vaginal flora—45	Fluomizin^®^ vaginal tablets 6 days	Disturbed vaginal flora in 44 women became healthy, only 1/45 patient had complication after surgery.	None
Grishchenko et al., 2006 [104] Open	66 (pregnant): BV—66	Fluomizin^®^ (34) vs. povidone-iodide vaginal	Sanitation of vagina, demonstrated favourable pregnancy conditions and perinatal outcomes.	None
Grishchenko et al. 2006 [105]	76: Mixed infection—76	Fluomizin^®^ (45) vs. chlorhexidine bigluconate vaginal	Cure rate after the treatment with Fluomizin^®^—96.7 %, with chlorhexidine bigluconate—66.0 %.	Local ADRs—5 patients; discontinued therapy—2
Demina et al., 2005 [103] Open	60 (pregnant): BV and ‘opportunistic flora vaginitis’—60	Fluomizin^®^ (32) vs. povidone iodine vaginal	Vaginal ecosystem recovery—in all pregnant women of group I after one treatment, and vaginal clearance of degrees I and II was seen in 91.7 %. No Candida albicans after Fluomizin^®^.	None
Petersen, 2002 [26] Controlled	121: BV—48; VVC—23; TV—5; other—45	Fluomizin^®^ vaginal tablets vs. povidone iodine vaginal	Symptoms decreased in the Fluomycin^®^ group from 5.13 to 1.33. BV: 96 % and 82 % of women were diagnosed BV-negative after therapy at short- and long-term follow-up, respectively. VVC: symptoms decreased from 6.5 at to 1.1. Flour vaginalis: symptoms decreased from 5.0 to 1.3.	5.8 % reported a local ADRs
Schmidt, 2000 (unpublished) Open, drug utilisation trial	446 (60 pregnant) BV—126; TV—10; VVC—97; flour vaginalis—213	Fluomycin^®^ N (10 mg DQC) vaginal tablets, daily 6 days	303 patients (67.9 %) at entry showed 3 to 4 symptoms, whereas at control only 42 patients (9.4 %). A distinct improvement in all documented clinical symptoms.	None
Strecker, 1993 [28] Open	388 (mixed group is included twice) BV—375; VVC—113	Gyken^®^ ovula twice (3 or 4 days interval)	Negative microscopy (cure): BV—115 (31 %); VVC—86 (76 %)	1 case of enhanced sleepiness
Warnecke, 1976 [101] Open	100 fluor vaginalis—7; ‘non-infective colpitis’—80; other—13	Eriosept^®^ 0.1 % vaginal spray once daily 3 days (84), 6 days (11)	Negative microscopy (cure): Fluor vaginalis—4 (57 %); ‘non-infective colpitis’—67 (84 %); other—10 (77 %)	None
Fauner, 1974 [91] Open	55 (pregnant) VVC: 55	Dequavagyn^®^ depot once, eventual repetition after 1 week	Negative microscopy (cure): VVC first therapy—46 (84 %), second therapy—8 (14 %)	None
Kucera, 1973 [93] Active-controlled	150: Bacterial fluor vaginalis—116; VVC—15; TV—14; mixed infection—5	Dequavagyn^®^ depot alone or with 0.0015 % estriol, or with 0.02 % estriol (Oestro-Dequavagyn^®^) twice, repetition after 1 week	Negative microscopy (cure, Dequavagyn^®^ depot alone): flour vaginalis—30 (79 %), VVC—3 (75 %), TV—4 (67 %), mixed infection—0 (0 %)	Not assessed
Kolbe, 1972 [92] Open	124: Bacterial fluor vaginalis—42; VVC—50; TV—27; TV + VVC—5	Dequavagyn^®^ depot once, eventual repetition after 10 days	Cure (examination not specified): Bacterial fluor vaginalis—42(100 %), VVC—47 (94 %). TV—26(96 %), TV +VVC—3 (60 %)	1 case of generalised pruritus
Mühlbauer, 1972 [97] Open	134: Bacterial fluor vaginalis—44; VVC—78; TV—12	Dequavagyn^®^ depot once (continuous DQC release over 8-10 days)	Negative microscopy (cure): Bacterial fluor vaginalis—33 (76 %), VVC 56 (72 %), TV—2 (24 %)	None
Tatra, 1972 [100] Open	311: Bacterial fluor vaginalis—180; VVC—69; TV—48; TV + VVC—13	Dequavagyn^®^ depot once, eventual repetition after 10 days	Negative microscopy (cure): Bacterial fluor vaginalis—147 (82 %), VVC—53 (77 %), TV—8 (30 %), TV + VVC 0 (0 %)	1 case of generalised pruritus
Lange, 1971 [94] Open	82: Bacterial fluor vaginalis—8; VVC—38; TV—36	Dequavagyn^®^ depot once, eventual repetition after 10 days	Negative microscopy (cure): Bacterial fluor vaginalis—8 (73 %), VVC—38 (76 %), TV - 36 (92 %)	1 case of purulent discharge
Martin & Martin, 1971; Martin, 1971 [34, 96] Open	200 (several indications possible): Bacterial fluor vaginalis—44; VVC—173; TV—4; ‘super-infection’—21	Dequavagyn^®^ depot once, eventual repetition after 10 days	Negative microscopy (cure): Bacterial fluor vaginalis—9 (66 %), VVC—117 (68 %), TV—2 (50 %), ‘super-infection’—19 (90 %)	1 case of pruritus (allergy)
Atlante, 1959 [88] Open	60 (4 mixed): VVC—2; TV—60	Dequadin^®^ pessary twice daily 5–6 days, repeated after menstruation	Negative microscopy (cure): VVC—4 (67 %), TV—54 (90 %)	None
Levinson, 1959 [95] Open	43: TV—43	Dequadin^®^ pessary twice daily 4 weeks	Negative microscopy (cure after 10 days): TV—17 (40 %)	Not assessed
Roddie, 1958 [99] Open	70: Bacterial fluor vaginalis—30; VVC—4; TV—36	Dequadin^®^ pessary twice daily 2 weeks	Symptoms after 2 weeks (absent/improved): bacterial fluor vaginalis—16 (59 %)/4 (15 %), VVC—2 (67 %)/1 (33 %), TV—1 (3 %)/5 (14 %)	None
Combination(s)				
Rippmann, 1974 [98] Open	106: VVC—26; TV—31; mixed infection—49	Oestro-Dequavagyn^®^ (DQC + 0.02 % estriol) twice, repetition after 1 week (continuous DQC release over 8–10 days)	Negative microscopy (cure): VVC—18 (69 %), TV—11 (35 %), mixed infection—39 (80 %)	none

### Treatment of bacterial vaginosis (BV)

Two recent high quality clinical studies (Weissenbacher et al. and Petersen et al.) have investigated efficacy and safety of 10 mg DQC vaginal tablets for treatment of BV—a total of 212 women have been treated [26, 32].

DQC have been used for a very long time and the first reference was from 1959. There was no consensus of the diagnosis of BV at that time. Hence, many older clinical data should be evaluated with scientific precaution. Nevertheless, additionally some older studies could also provide some supportive data on clinical efficacy of DQC in the treatment of vaginal infections which currently could be described as BV. Within these older clinical studies (including some unpublished data) a total of 577 women with more or less well diagnosed BV-like syndromes have been treated with various vaginal DQC preparations [26, 28, 32, 104, 107].

In a recently published multicentre, controlled study Weissenbacher and colleagues compared the efficacy of DQC 10 mg vaginal tablets (Fluomizin^®^) once daily for 6 days to clindamycin vaginal cream 2 % for 7 days in the treatment of BV [32]. To qualify for inclusion, women had to be diagnosed with BV, for which all 4 Amsel criteria had to be present. Follow-up visits were performed at 1 week and at 4 weeks after the treatment. A total of 321 women were randomised to receive either 10 mg DQC (*n* = 164) or clindamycin (*n* = 157). The primary efficacy outcome was clinical cure, where clinical cure was defined as the absence of clue cells and a negative result for at least 2 other Amsel criteria. Weissenbacher et al. have used the more than 20 % clue cells criterion: to be cured of BV the patient could have clue cells present but less than 20 %. At week 1, clinical cure rates were 81.5 % in women treated with 10 mg DQC and 78.4 % in clindamycin-treated women, the difference was not statistically significant. The treatment of BV with a 6-day course of 10 mg DQC vaginal tablets had therefore equal efficacy as a 7-day course of clindamycin vaginal cream. At week 4, the overall clinical cure rates observed were 79.5 % with 10 mg DQC and 77.6 % with clindamycin, demonstrating that the efficacy of DQC was as good as clindamycin (statistically significant) also at long-term follow-up. Most Amsel criteria were fairly comparable with slight, but clinically not relevant, differences between the groups. The total failure rates, including non-responders and BV recurrences, for the two groups were also comparable. The number of women with positive cultures of *Candida* spp. (‘colonisation’) at week 4 was slightly higher in the clindamycin group (14.6 %) than in the DQC group (9.3 %), however, due to small figures statistically not significant. Consistent with these results, symptomatic VVC at second follow-up was half as common with DQC (2.7 %) than with clindamycin (5.8 %). No serious adverse drug reactions (ADRs) were observed. The proportion of women reporting ADRs was 17.8 % in the DQC group and 20.3 % in the clindamycin group. The most frequently reported reactions in both groups were vaginal discharge followed by vulvovaginal pruritus.

Petersen et al. [26] in a controlled, randomised, double-blind, multicentric clinical study compared the efficacy and safety of 10 mg DQC vaginal tablets (Fluomizin^®^) and 200 mg vaginal povidone iodine in a parallel-group design [26]. A total of 180 women with vaginal infections of varying aetiology participated in this study. Women were randomly allocated to one of the two treatment groups (DQC—121 and povidone iodine—59) and were treated once daily for 6 days. The total symptom score was defined as the overall primary efficacy parameter. Based on this parameter, the study demonstrated therapeutic equivalence of vaginal DQC and the reference preparation. In the BV subgroup a total of 73 women were included, whereof 48 were treated with DQC. Using Amsel criteria, 96 % (after 1 week) and 82 % (after 4 weeks) of patients were cured after 10 mg DQC therapy. The percentage of women with positive cultures of *G. vaginalis* decreased clearly from 60 % before to 7 % after treatment. The improved microbial status was also demonstrated by the increased percentage of women with a healthy degree of purity (pure lactobacilli flora) in the DQC group (at entry—0 %, at follow-up 1 (after 1 week)—37 %, at follow-up 2 (after 4 weeks)—46 %). The normalisation of the healthy flora was faster in the DQC group as compared to the reference group (at entry—0 %, at follow-up 1–14 %, at follow-up 2–50 %).

Strecker and colleagues in a non-controlled clinical study tested the efficacy and local tolerability of DQC in a total of 388 women whereof 274 were diagnosed with BV [28]. Treatment with a vaginally administered 20 mg DQC ovula (2 ovules daily in a 3 days interval) was successful in 70–85 % of the patients with BV with/without accompanying candidosis and with VVC alone. The therapeutic efficacy of was rated as good or very good by 91 % of the patients and by 99 % of the investigators.

Radzinsky and co-investigators [107] presented the results of a multicentre trial using different therapy regimens in 640 non-pregnant women of reproductive age with bacterial vaginal infections different aetiology. The trial was divided into two stages: In the first stage, women received anti-infective therapy against bacterial vaginosis or nonspecific vaginitis; afterwards restoration of the vaginal ecosystem was investigated with probiotic (outcomes are not described here). In the first stage, women received vaginal anti-infective treatment at the physician’s discretion (DQC, clindamycin, chlorhexidine, povidone iodine, or metronidazole/miconazole combination). In the group with BV, 409 women positive for BV according to the Amsel criteria were included in the statistical analysis. The efficacy of vaginal DQC based on Amsel criteria (*n* = 144, 77.8 %) was equivalent to the efficacy of the other anti-infective therapies (*n* = 281, 77.1 %).

Overall the clinical studies have demonstrated the excellent efficacy of 10 mg DQC formulation Fluomizin^®^ in the treatment of BV. Clinical cure rates of 10 mg DQC were equal to clindamycin 2 % vaginal cream, one of the current ‘gold standards’ in the treatment of BV. Furthermore, vaginal pH, number of lactobacilli and clinical symptoms improved clearly during 10 mg DQC therapy; and additionally DQC demonstrated trend to less cases of post-treatment VVC than after clindamycin therapy. The drug was well tolerated; no serious ADRs were observed.

### Treatment of vulvovaginal candidosis (VVC)

Dequalinium chloride in different vaginal formulations has been used to treat a total of 653 women with VVC, and cure rates were reported between 70  and 90 % [26, 28, 34, 88, 91–94, 96–100].

In a series of clinical studies [34, 91–94, 96, 97, 100], altogether 490 patients with VVC were treated with a DQC 20 mg depot preparation. In 70–80 % of the cases, patients were cured. Kolbe [92] reported even a cure rate of 94 %. In 15–20 % of the cases, symptoms improved and the microscopic analysis of *C.* *albicans* showed a clearly reduced yeast load. However, in 10–25 % of the cases, even a repetition of the treatment was not successful [34, 93]. Also in pregnant women with VVC (*n* = 55), DQC depot preparation led to complete cure in 84 % of the cases [91].

Strecker et al. [28] tested a DQC 20 mg vaginal ovula at day 1 and one ovula at day 4 in women suffering from VVC with (*n* = 103): a positive effect—cure or improvement—was demonstrated on more than 90 % of cases.

A DQC 20 mg and estriol combination resulted in total disappearance of *C. albicans* in 69 % of the microscopic evaluated vaginal smears and in considerable reduction of *C. albicans* in 19 % of the vaginal smears [98]. In some studies sexual partners were also parallelly treated with a DQC vaginal cream (currently commercially not available) [97, 100]: the cure rate remained at a similar range 77–91 %.

In the earlier mentioned controlled, multicentric clinical trial, Petersen with collaborators compared the efficacy and safety of 10 mg DQC vaginal tablets (Fluomizin^®^) and 200 mg vaginal povidone iodine in a parallel-group design also in women with VVC [26]. Of the total of 180 women, 35 women with VVC (23 women in DQC and 12—in povidone group) were included, where VVC was diagnosed by its typical signs and symptoms and by the presence of yeasts in the wet mounts or in cultures. The clinical symptoms improved clearly from a total score of 6.5 at entry to 1.1 at the second follow-up, if treated with DQC. At entry, all patients (100 %) were positive for *Candida* spp. The percentage of positive patients decreased in the DQC group clearly to 21.7 % at follow-up 1 and 15.8 % at follow-up 2; the corresponding values in the reference group were 33.3 and 18.2 %. Global efficacy of DQC at the end of the study was rated as complete or considerable improved in 84 and 89 % of cases by investigators and patients, respectively. DQC therapy was well tolerated with no serious ADRs observed.

Treatment in a so far unpublished multicentre drug utilisation trial by Schmidt has assessed the effectiveness and tolerability of DQC 10 mg vaginal tablets (Fluomizin^®^) in the treatment of ‘acute colpitis’ (vaginal infections) under conditions of daily practice. In the VVC group 97 women were included from 30 centres in Germany. Whereas 86.6 % of the women had three or four symptoms at the entry examination, this was the case for only 12.2 % at the control examination. The number of women with Candida-positive culture before therapy was 100 %, and decreased to 20 % after the DQC treatment. No ADRs were observed.

Overall, different vaginal formulations of DQC in women with symptomatic VVC in clinical studies demonstrated similar good efficacy (cure and/or improvement rates) and safety. With the DQC 10 mg vaginal tablets Fluomizin^®^ formulation a total of 120 women (including unpublished) with VVC have been treated demonstrating the high clinical efficacy and tolerability also for this vaginal formulation [26].

### Treatment of less defined vaginal infections

Even though that there are concern that vaginal infections are also caused by aerobic pathogens, there is still no consensus of how ‘fluor vaginalis’ should be diagnosed and it is difficult to compare the results from different investigators. DQC in different vaginal formulations has been also used for the treatment of the following less defined vaginal infections: aerobic bacterial vaginal infections (AV, AVF, bacterial ‘fluor vaginalis’, etc.), mixed vaginal infections and co-infections, and Trichomoniasis/TV. Again, the classification of mixed vaginal infections and/or co-infections is still purely described. These facts are not allowing to draw sufficiently clear scientific evaluations but some general conclusions probably still could be done.

#### Aerobic bacterial vaginal infections

Within 11 clinical studies, a total of 968 women that have been diagnosed with this type of vaginal infections were treated with vaginal DQC [26, 34, 92–94, 96, 97, 99–101, 103, 105, 107]. Roddie [99] has reported treatment results with DQC 10 mg pessaries twice daily in 30 women with ‘bacterial fluor vaginalis’. Symptoms improved after 2 weeks and resumed in 70 % of the cases after 3 weeks of treatment. The therapy did not lead to irritation or sensitisation of the vagina.

Different clinical trials reported good results in treatment of ‘bacterial fluor vaginalis’ with a DQC depot preparation that, once inserted into the vagina, releases continuously DQC for 8–10 days. A single treatment with 10–20 mg DQC (the precise dose is not mentioned in the publications) cured 66–76 % of the 137 patients [34, 92–94, 96, 97, 108].

In the above-mentioned multicentre drug utilisation trial, Schmidt in an unpublished trial have assessed the efficacy and safety of DQC 10 mg vaginal tablets in the treatment of ‘acute colpitis’ (poorly defined vaginal infections). Totally 213 women were included in the subgroup of MV (bacterial mixed infection, ‘fluor vaginalis’). Whereas 62.9 % of the women had three or four symptoms at the entry examination, this was the case for only 10.0 % at the follow-up examination. The number of women with positive cultures for gram-negative and gram-positive bacteria decreased approximately by 50 %. No ADR was observed.

Petersen [26] and colleagues includes in their clinical study also 73 women diagnosed with ‘fluor vaginalis’ (aerobic bacterial vaginal infection), where signs and symptoms of vaginitis and a disturbed vaginal flora were present, but BV, VVC and TV were excluded. The total symptoms score decreased under therapy with DQC from 5.0 ± 1.9 at entry to 1.9 ± 1.5 at follow-up 1 and 1.3 ± 1.3 at follow-up 2. The number of leukocytes (>10 per field of view) decreased in the DQC group for 42.2 % of patients at entry to 9.3 % at follow-up 1 and 8.6 % at follow-up 2 (4 weeks). Furthermore, the DQC therapy resulted in a decrease of the positive cultures for *Streptococcus* ssp., *Enterococcus* spp., and *E.* *coli* by 36, 49, and 73 %, respectively, at the end of the study.

Radzinsky and colleagues [107] investigated in their above-mentioned clinical study also the efficacy of vaginal DQC in 213 women with ‘nonspecific vulvovaginitis’ presenting a clinical picture of an inflammatory and intense leukocyte reaction (>20 leucocytes field of view in the vaginal smear, magnification 400 times). The efficacy in the group of the patients receiving DQC (*n* = 67) was estimated above 76 %. The anti-infective efficacy of the broad spectrum DQC was found to be similar to that of other most commonly used agents in treating vulvovaginal infections.

All clinical studies consistently reported a good clinical efficacy of DQC in the treatment of aerobic bacterial vaginal infections. This is particularly interesting as for this pathology no standard treatment has been established yet. The therapy with vaginal DQC resulted in a clear reduction of the clinical symptoms, reduction of presence of relevant facultative-pathogenic microorganisms in vaginal cultures, and was well tolerated.

#### Mixed vaginal infections

Clinical studies including women with MV are rare, only 5 published studies report the results of a total of 179 women [28, 92, 93, 98, 100]. Recently Sobel et al. [14] described the MV as an infection with at least 2 pathogens that are well defined. Additionally, it is important to understand that ‘mixed flora’ and ‘mixed vaginal infection’ as defined by Sobel could not always be the same nosologic entity.

After vaginal administration of an DQC ovule 20 mg at day 1 and one DQC ovule at day 4 to patients suffering from VVC with BV co-infection (*n* = 11), approximately 1/3 of the patients showed improvement of symptoms, and 2/3 of the patients were completely cured. These findings were confirmed by the significant reduction of the vaginal pH value below 4.5 in approximately 2/3 of the cases. Furthermore, the positive amine test turned into negative after the treatment in 87 % of the women with BV co-infection. Therapy was not successful in only 1/10 of the cases [28].

The therapy with a combination of DQC (applied dose not known) with 0.0015 % estriol (Oestro-Dequavagyn^®^) of 49 women with MV resulted in total disappearance of the mixed flora in 80 % of the vaginal smears, and in considerable reduction of the mixed flora in 4 % of the cases [98].

#### Trichomoniasis

Within clinical studies, a total of 329 women with TV have been treated with different vaginal DQC preparations [26, 34, 88, 92–97, 99, 100].

In a series of clinical studies, the efficacy and tolerability of DQC 10 mg pessaries was assessed in altogether 139 trichomoniasis cases. The average treatment lasted 6 weeks and pessaries were applied 1–2 times per day. Overall, in 40–90 % of the cases *T.* *vaginalis* was eradicated and patients were cured, and 10–60 % showed considerable improvement of symptoms [88, 95, 99]. In one study, treatment was even successful in women who had previously failed to respond in any satisfactory degree to any other forms of treatment including the use of pessaries containing oxytetracycline which today is no first line medication. However, a number of cases relapsed in a period of 2–16 weeks after treatment end [100]. The treatment of trichomoniasis with DQC seems to be less efficient—the overall efficacy in this disease was reported 17–50 % [34, 96, 97]. In order to achieve complete treatment success concomitant systemic medication with a trichomonacidal agent, for example metronidazole, should be used [34, 92–94, 100].

### Clinical safety

Within clinical studies, different vaginal DQC preparations have been used to treat more than 3000 women with various vaginal infections. The overall share of ADRs was with 2.4 % very low, showing the excellent local tolerance of vaginally administered DQC. With the DQC 10 mg vaginal tablets Fluomizin^®^ formulation, 1224 women have been treated in controlled or open clinical studies and only 95 ADRs (7.8 %) were observed and none of them were serious [26, 32, 102–106]. The majority of the ADRs in clinical studies (about 90 %) were local reactions, particularly vaginal discharge, vulvovaginal pruritus, and a vaginal burning sensation.

#### Clinical studies in pregnant women

A total of 181 pregnant women have been intentionally treated with vaginal DQC in four clinical studies, and no adverse effects on pregnancy or on the health of the foetus/new-born child (based on pH, Apgar scale, and follow-up until 1 year) have been observed.

Fauner and Binder [91] treated 55 pregnant women with VVC by a DQC depot preparation that continuously releases active substance over 8–10 days. The tolerability and safety of the treatment was excellent and no ADRs were reported on pregnant women and new-born.

In an unpublished drug utilisation study, 60 pregnant women were treated with DQC10 mg. The tolerability of the treatment was ‘very good’ or ‘good’ for 95 % of the patients and did not differ from that of the entire group of patients. No ADRs were reported. Demina and colleagues [103] investigated the efficacy and safety of DQC vaginal tablets (Fluomizin^®^) in the treatment of bacterial vaginal infections during pregnancy. Sixty women in the first trimester of pregnancy at risk for miscarriage and thirty healthy pregnant women without complications were included. The latter group of healthy pregnant women served as reference group. The women at risk for miscarriage either received Fluomizin^®^ vaginal tablets (group I: *n* = 32) or a drug containing povidone iodine (group II: *n* = 38). No ADRs were observed. In the DQC group, no case of spontaneous abortion was observed, whereas in the povidone iodine group 2 spontaneous abortions were observed and any treatment by iodine during pregnancy presents a risk on the foetal iodine metabolism.

Grishchenko et al. [104] treated 34 pregnant women (week 6–8) suffering from BV with DQC 10 mg vaginal tablets (Fluomizin^®^) once daily for 6 days. A control group of 32 pregnant women was treated with povidone iodine. Following this treatment, the course of the pregnancy, the pre-natal condition of the foetus and perinatal pathology were studied. Placental dysfunction in week 16 of pregnancy occurred in fewer patients treated with DQC (29.4 %) than in the control group (84.4 %). The greatest differences between the groups were found in the amount of amniotic fluid and placenta maturity. Although the precise role of BV in pregnancy is still debated, the authors concluded that BV is accompanied by the development of placental dysfunction, causing high levels of pre-natal foetal hypoxia and perinatal pathology, and the effective treatment of BV with DQC in the first trimester of pregnancy could improve the perinatal outcomes [84, 91, 103, 104].

## Summary and conclusions

Vaginal infections are a common medical problem and are responsible for a large proportion of gynaecological outpatient visits. These infections are associated with a variety of serious adverse health outcomes, especially in pregnant women. The most common vaginal infections are BV and VVC; however AV/AVF and mixed infections are also considerably frequent. In clinical practice the correct diagnosis of vaginal infections, particularly mixed infections, is often difficult and finding an effective treatment can be challenging for the clinician.

The main treatment objectives are the alleviation of the symptoms, the elimination of pathogens, and eventually the recovery from disturbed to a healthy lactobacilli-dominated vaginal flora. Additionally high tolerability of the treatment is needed to increase the compliance and acceptability by the women. However, treatment options for the vaginal infections are limited, and the outcome is often unsatisfactory. Even though current treatments show acceptable short-term efficacy with cure rates of 70–80 %, recurrent vaginal infections as well as post-treatment candidosis remain an issue.

Acquired microbial resistance and spread of intrinsically resistant microorganisms as well as bacterial biofilms have to be considered future challenges for an effective treatment of vaginal infections.

Antibiotics (typically metronidazole and clindamycin) and antimycotics (azoles) are the most recommended treatments. In terms of tolerability and safety, vaginal therapies usually have fewer side effects than oral products. Increasing resistances and post-treatment vaginal infections (candidosis after antibacterial therapy, bacterial infections after antimycotic therapy) also have an impact on the outcome of the therapy. Due to the risk of vaginal infections causing serious complications in pregnancy, safe therapies for pregnant women are also of great importance. Thus alternative treatments are a key medical need in the management of vaginal infections.

DQC is an anti-infective agent belonging to the class of quaternary ammonium compounds. Its broad antimicrobial activity against all relevant vaginal pathogens is well established demonstrated by several investigators over the past decades. DQC exhibits a fast and strong bactericidal and fungicidal action. The primary mode of action of DQC is based on an increase in cell permeability with the subsequent loss of enzyme activity. Resistance of pathogens is unlikely due to its multiple mode of action and has not been reported. The vaginal application of DQC results in high local concentration, but due to low absorption systemic exposure is negligible.

In clinical studies described in the literature, medicinal products with DQC of different concentrations and vaginal formulations were used. The efficacy over the entire dose range was similar and no difference in the tolerability was described. Local treatment of vaginal infections with DQC is well established, and many publications have demonstrated its clinical efficacy in different vaginal infections. Overall, more than 3000 women have been treated with vaginal DQC in clinical studies for the following vaginal infections: BV, VVC, AV/AVF, trichomoniasis and mixed vaginal infections. Independent of the specific DQC drug used, treatment of vaginal infections of bacterial and mycotic origin has been shown to result in similarly high cure rates (70–90 %). Furthermore, a recent clinical study with DQC also confirmed the comparable efficacy with clindamycin in the treatment of in BV. The DQC treatment is a very interesting treatment option for BV but as there are no long follow-up data available, it would be still reasonable to perform more clinical studies investigating cure rates and at least one clinical study with longer follow-up (6 months).

The safety of DQC has also been demonstrated in these various clinical studies. No serious ADRs have been observed. The majority (>90 %) of ADRs were local reactions (particularly vaginal ulcerations, vaginal discharge, vulvovaginal pruritus, and vaginal burning sensation) which were mostly mild or moderate in intensity. Due to the negligible absorption of DQC, systemic events have been rarely reported. Furthermore, the available clinical data suggest the suitability of DQC for use during pregnancy also. Vaginal DQC is also listed in the German guideline ‘Bacterial vaginosis’ as well as in the Portuguese guidelines for treatment of vaginal infections as an effective treatment for BV [31].

From a clinical point of view DQC could offer an attractive treatment for vaginal infections, both for BV and VVC as well as in case of mixed infections—this treatment concept should be further investigated in broader clinical studies. Vaginal DQC has been shown to have equal clinical efficacy as clindamycin in the treatment of BV and to be well tolerated with negligible systemic effects. It has the major advantage that its broad antimicrobial activity makes it appropriate for the treatment of mixed vaginal infections and in case of uncertain diagnosis. Moreover, resistance of pathogens is unlikely due to its multiple mode of action, and vaginal DQC provides also a reduced risk for post-treatment vaginal infections of different aetiology that is quite frequent after specific antibacterial or antimycotic treatment.

In conclusion, the current formulation DQC 10 mg vaginal tablets (Fluomizin^®^) as 6-day therapy with its broad antimicrobial spectrum and excellent tolerability offers a safe and effective option for empiric therapy of different vaginal infections in daily practice.
